# Supplementary material for: Unconventional fractional quantum Hall states and Wigner crystallization in suspended Corbino graphene
Source: Nat Commun. 2018 Jul 17;9:2776. doi: 10.1038/s41467-018-05094-8 (PMC6050265; doi:10.1038/s41467-018-05094-8)
Supplement: Supplementary file 1 — SI [file 41467_2018_5094_MOESM1_ESM.pdf]

Unconventional fractional quantum Hall states and Wigner crystallization in suspended Corbino graphene

Manohar Kumar[1,2], Antti Laitinen[1], and Pertti Hakonen*[1]

[1]Low Temperature Laboratory, Department of Applied Physics, Aalto University, Espoo, Finland
[2]Laboratoire Pierre Aigrain, Département de Physique de l'École Normale Supérieure -PSL Research University, CNRS Université Pierre et Marie Curie-Sorbonne Universités Université Paris Diderot-Sorbonne Paris Cité, Paris, France

*To whom correspondence should be addressed; *pertti.hakonen@aalto.fi*

In this supplement for the manuscript "Unconventional fractional quantum Hall states and Wigner crystallization in suspended Corbino graphene" we provide additional information on sample fabrication, current annealing of graphene to enhance mobility, sample characterization, the identification of fractional states using transconductance, transport mechanisms in our samples, rf-measurements of Wigner crystal, and the nature of its pinning.

Supplementary Note 1: Sample Fabrication

Our sample fabrication is based on well-chosen combination of resists with differential selectivity, which allowed the fabrication steps for the deposition of a suspended top contact. In this work, we employed heat assisted mechanical exfoliation technique [1]. The application of heat assisted exfoliation on top of LOR resist produced large graphene flakes, which was the basic requirement for a good Corbino device. A $250+250$ nm thick base layer of LOR-3A [2] was spun in two steps on SiO_2/Si++ and baked at 200°C for 5 and 12 min, respectively. A graphite covered tape was pressed against the LOR-covered wafer for 1 min and subsequently baked on a hot plate at 100°C for 2 min. The tape was peeled from the chip after it had cooled down to room temperature. The exfoliated graphene flake was first characterized optically and then using Raman spectroscopy at 633 nm.

In the first lithography step, the lead for the outer ring and its contact pad were fabricated. A double layer PMMA (950K/A3: top layer and 50K/A11: bottom layer) spun and patterned using e-beam. A Cr/Au sandwich of $5/70$ nm thickness was evaporated at pressure $p < 6 \times 10^{-8}$ mBar. After metal deposition, special care was taken during the metal lift-off process: the lift off was performed in 80°C xylene and the unwanted residual metal from the circular hole of outer lead was removed using micromanipulation. The outer ring electrode is of rectangular geometry extending over the graphene flake with a circular hole of diameter $2-10$ μm, see Supplementary Fig. 1c. On the processed sample, another 500 nm thick LOR was spun. A via in the center of the circular hole of outer lead was opened in the top LOR layer using e-beam lithography. A dose of 1000 μC/cm^2 was used for this step. The exposed pattern was developed in ethyl lactate for 60 s and rinsed in hexane. The diameter of the via varied between $0.5-5$ μm, see Supplementary Fig. 1d. This via acts as foundation for the inner electrode contact in the concentric Corbino geometry. For fabrication of the inner lead, a double layer PMMA of 200 nm was spun and patterned using e-beam lithography. On the patterned device, Cr (5 nm)/Au (100 nm) was deposited, and the lift off was done in hot xylene with a rinse in hexane, see Supplementary Fig. 1e.

In final steps of the lithography, the graphene between inner and outer ring leads was suspended by exposing LOR to a dose of 1000 μC/cm^2. In order to expose the outer lead bonding pad, LOR on the larger structures was exposed to an e-beam dose of 700 μC/cm^2. The device was developed and cleaned in ethyl lactate for 60s and rinsed in hexane for 60s. A schematic cross section of the final device is depicted in Supplementary Fig. 1f and an SEM image of a complete sample structure is shown in Fig. 1 of the main text. For bonding, part of the pad was pressed against the SiO_2, as shown in Supplementary Fig. 1g. These pressed pads were used for bonding. The finished devices were electrically characterized at room temperature, and devices with low resistance were selected for low temperature measurements.

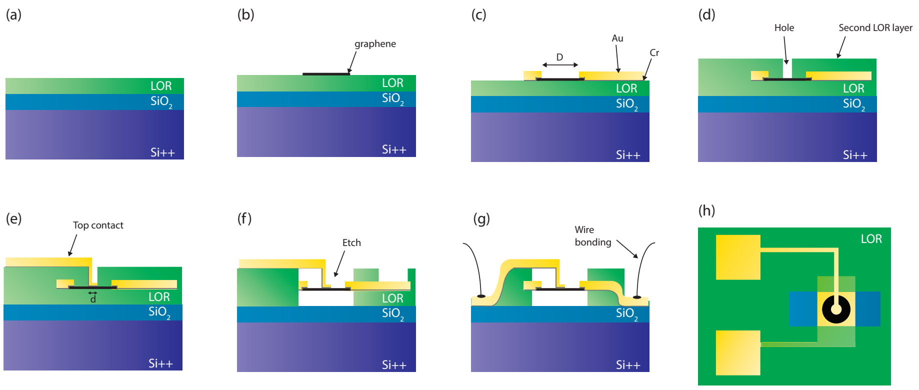

Supplementary Figure 1: Fabrication process steps: **a)** LOR layer with thickness of ~500 nm is spun on a cleaned Si/SiO_2 chip. **b)** Mechanical exfoliation of graphene on top of the LOR layer. **c)** Fabrication of outer lead and its bonding pad. **d)** Formation of a via for the inner lead using a second LOR layer. **e)** Air bridge connecting inner lead and pad is patterned using an e-beam lithography step with PMMA. **f)** Suspension of graphene between the inner and outer leads and uncovering of the outer lead-pad by e-beam exposure. **g)** Status after wire bonding: gold pads are deformed and pushed partly down to silicon. **h)** A schematic top view of a sample with suspended top contact: black color shows suspended graphene, blue color shows the exposed window in LOR, light yellow color shows the covered outer lead and bright yellow color indicates clean gold of the inner lead and the outer leads.

Supplementary Note 2: Sample characterization

Our measurements down to 20 mK were performed in a BlueFors LD-400 dilution refrigerator. The measurement lines were twisted pair phosphor-bronze wires supplemented by three stage RC filters with a nominal cut-off given by $R = 100$ Ohms and $C = 5$ nF. However, due high impedance of the quantum Hall samples the actual cutoff is determined by the sample resistance. For magnetoconductance measurement, we used an AC peak-to-peak current excitation of 0.1 nA at $f = 3.333$ Hz.

For transconductance $g_{\mathrm{m}} = dI/dV_{\mathrm{g}}$ we measured both magnitude Mag$\{g_{\mathrm{m}}\}$ and phase Arg$\{g_{\mathrm{m}}\}$ using low frequency lock-in detection. The best results for g_{m} correlation analysis were obtained by recording Arg$\{g_{\mathrm{m}}\}$ at a bias voltage V that corresponded to the onset of the V^{α} regime. Consistent information was obtained by analyzing the simultaneously acquired Mag$\{g_{\mathrm{m}}\}$. The gate frequency in AC transconductance measurements was set at f = 17.777 Hz, while the peak-to-peak AC excitation amplitude was adjusted to correspond to charge of one electron over the sample. The DC bias between source and drain was varied in the range V = 0.1 - 0.5 mV.

Annealing

Our suspended Corbino graphene samples were predominantly of p-type. During typical annealing process, the doping shifts to n-type before reaching the desired charge-neutral state. For achieving charge neutrality, a high current annealing was performed. We employed voltage bias for our current annealing process: In contrast to current-biased devices, voltage-biased samples are protected against possible rise in resistance due to shifting of the Dirac point. A typical bias voltage value used in annealing of our samples was 1.6 ± 0.1 V; in spite of strong heating of the refrigerator, the sample was kept at temperatures $T < 1$ K during annealing. Slight asymmetry in the concentricity of the electrodes in our Corbino geometry, inverse radial decay of current, and local variation of the contact resistance result in non-uniformity of current annealing. Owing to this non-uniformity, our suspended Corbino disk becomes split into differently annealed graphene domains, each having their own charge neutrality point. This can be seen in Supplementary Fig. 2a. Eventually, during annealing process several of these peaks merge to give a single Dirac peak or closely degenerate Dirac peaks. See Supplementary Fig. 2b for the final R_{d} vs V_{g} curve of the sample EV* after annealing.

Residual Doping

The level of residual charge doping was extracted from G vs n -plots on *loglog*-scale as the point where conductance G levels off when approaching the Dirac point. We employed straight line fits (power laws with exponent around 1/2) to the conductance data in order to better define the position of this cross-over point, as shown in Supplementary Fig. 2c. The residual doping for other samples was extracted in the same way: $n_{0,\mathrm{XD}} = 2 \times 10^{10}$ cm^{-2} and $n_{0,\mathrm{EV}} = 6 \times 10^{9}$ cm^{-2}.

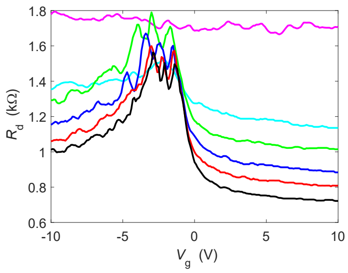

(a) Annealing

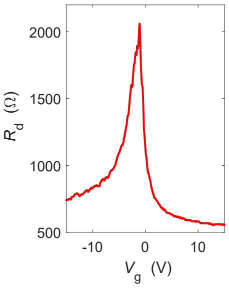

(b) Single gatesweep

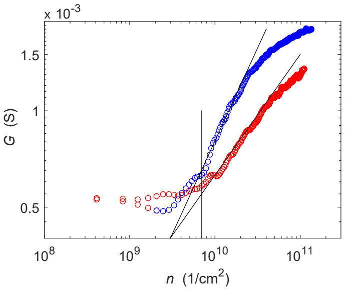

(c) Residual charge density

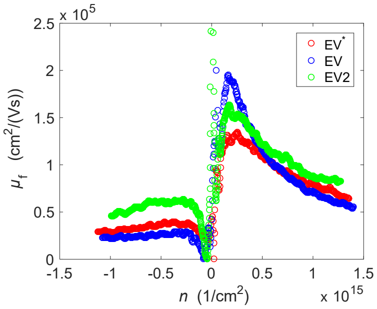

(d) Electron mobility

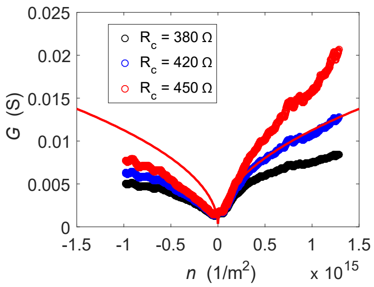

(e) Contact resistance

Supplementary Figure 2: Sample characterization : **a)** Annealing progress of a Corbino sample with outer diameter $D = 3.2\ \mu$m and inner contact diameter $d = 0.8\ \mu$m. The magenta curve at the top is the first differential resistance R_d trace measured after annealing current $I \approx 1.5$ mA ($V \approx 1.3$ V) while the black curve, obtained after $I \approx 2$ mA ($V \approx 1.7$ V), denotes the final annealing result (sample EV in Table I of the main paper). **b)** R_d vs V_g curve for sample EV* after annealing. **c)** G vs n for sample EV* on *loglog*-scale: red and blue circles represent data on the hole and electron sides, respectively. The black lines represent power law trends in the data. The cross-over point of the curves from power law to constant conductance is taken as n_0, which yields here $n_0 = 6 \times 10^9$ cm^{-2}. R_c was not subtracted in this analysis. **d)** Mobility as a function of electron density n ($n < 0$ corresponds to holes) determined for samples EV*, EV, and EV2 at zero magnetic field. Contact resistance R_c (see text) has been subtracted off from the measured resistance, *i.e.*, $G = \frac{1}{R_\mathrm{d} - R_\mathrm{c}}$ in this analysis. **e)** DC conductance measured for sample EV2 assuming an external series resistance, a contact resistance of $R_\mathrm{c} = 380\ \Omega$ (black circles), $R_\mathrm{c} = 420\ \Omega$ (blue circles), and $R_\mathrm{c} = 450\ \Omega$ (red circles). The solid red curve illustrates a theoretically calculated curve which agrees with the data when the contact resistance is set to $R_\mathrm{c} = 420$ Ω, *i.e.*, $G = \frac{1}{R_\mathrm{d} - R_\mathrm{c}}$ for the data points.

Supplementary Note 3: Mobility and Contact resistance

Field effect mobility of our samples was calculated using the formula

$$\mu_{\mathrm{f}} = \frac{\sigma - \sigma_0}{ne}, \tag{1}$$

where σ_0 is the measured minimum conductivity, and n denotes the charge carrier density. The conductivity in Corbino geometry can be calculated from measured conductance G using

$$\sigma = G\frac{\ln(r_{\mathrm{o}}/r_{\mathrm{i}})}{2\pi}, \tag{2}$$

where r_{o} and r_{i} are the radii of outer and inner contacts, respectively.

When calculating the mobility using Supplementary Eqs. 1 and 2, we take into account the contact resistance R_{c}. We estimate R_{c} from the comparison of measured data to the theory based on evanescent mode transport [3]. Supplementary Fig. 2e displays such a fitting for sample EV2, which yields R_{c} = 420 Ω for the contact resistance. Similar comparison was done for EV and EV*, and we obtained R_{c} = 410 Ω for EV* and R_{c} = 550 Ω for EV.

After taking the contact resistance into account, we obtain for the maximum mobility of our samples: $\mu_{\mathrm{f,XD}} \approx 5 \times 10^4$ cm^2/Vs, $\mu_{\mathrm{f,EV^*}} \approx 1.4 \times 10^5$ cm^2/Vs, $\mu_{\mathrm{f,EV}} \approx 2.0 \times 10^5$ cm^2/Vs, and $\mu_{\mathrm{f,EV2}} \approx 2.5 \times 10^5$ cm^2/Vs. These values are listed in Table I in the main paper. According to mobility, the quality of our samples follows the order EV2 $>$ EV $>$ EV* $>$ XD. Supplementary Fig. 2d illustrates the dependence of μ on the change density. The mobility on the hole side appears to be by factor of $3-5$ worse than on the electron side. We assign this difference to the presence of *pn* interfaces near the chromium-gold contacts which are known to have appreciable negative doping [4]. The *pn* interfaces reduce the conductance and result in a lowered apparent mobility for holes.

Contact Resistance

The full calculation of conductance through graphene with contact transparency is quite a tedious task [4]. Here we will describe in simple terms, how the contact resistance can be viewed as an additive quantity on top of the graphene conductance which arises for graphene Corbino disk from the Dirac equation [3].

Conductance through a double barrier graphene system

To derive the total transmission through the sample with contact resistance, we follow the procedure of Ref. [4]. First we note that usually phase coherence is

more sensitive to disorder than reflection and transmission, and we expect that the former is destroyed but the latter is not affected by disorder (ballistic regime). This assumption is supported by the experimental results on the Fabry-Pérot resonances which are found to be weak. Furthermore, when destroying the Fabry-Pérot resonances fully by an applied bias, the overall conductance does not change appreciably. Hence, we conclude that incoherent treatment of transmission probabilities is well justified in our sample.

In general, for the case of incoherent tunneling through a symmetric graphene barrier with equal transmission T for the left and right slopes, the total probability of transmission through the barrier is given by [4]

$$T_{\mathrm{tot}} = \frac{1}{2T^{-1} - 1}, \tag{3}$$

If a finite contact resistance exists, then a finite transmission probability $0 \leq T_{\mathrm{c}} \leq 1$ should be included in to the value of T in Eq. (3). By applying the incoherent addition model, we may write

$$T = \frac{T_{\mathrm{c}}|t|^2}{T_{\mathrm{c}} + |t|^2 - T_{\mathrm{c}}|t|^2}, \tag{4}$$

where $|t|^2$ denotes the transmission probability through the graphene sample and T_{c} gives the transmission at the contacts. This equation can be written in a more transparent form using resistances scaled by the quantum resistance $R_{\mathrm{Q}} = \frac{h}{2e^2}$: $|t|^2 = R_{\mathrm{ideal}}/R_{\mathrm{Q}}$, $T_{\mathrm{c}} = R_{\mathrm{c}}/R_{\mathrm{Q}}$, and $T = R/R_{\mathrm{Q}}$. Then we obtain

$$\frac{R}{R_{\mathrm{Q}}} = \frac{R_{\mathrm{ideal}}}{R_{\mathrm{Q}}} + \frac{R_{\mathrm{c}}}{R_{\mathrm{Q}}} - 1. \tag{5}$$

By identifying $\frac{R_{\mathrm{ideal}}}{R_{\mathrm{Q}}} - 1$ as the scaled sample resistance (zero in the ballistic limit), we find direct additivity of the contact resistance with the ideal, calculable resistance of the sample.

Extracting Contact Resistance

Transport properties of a graphene Corbino disk were calculated in Ref. [3]. The conductance is determined by

$$G = \frac{se^2}{h} \sum_{\mathrm{j}=\frac{1}{2},\frac{3}{2},\frac{5}{2}\dots} T_{\mathrm{j}}, \tag{6}$$

where $s = 8$ and T_j is the transmission coefficient of the jth channel. Transmission coefficient is given by

$$T_\mathrm{j} = \frac{16}{\pi^2 k^2 R_1 R_2} \frac{1}{(\mathfrak{D}_\mathrm{j}^+)^2 + (\mathfrak{D}_\mathrm{j}^-)^2}, \tag{7}$$

with

$$\begin{aligned}\mathfrak{D}_\mathrm{j}^\pm = \Im\{&H^{(1)}_{\mathrm{j}-1/2}(kr_\mathrm{i})H^{(2)}_{\mathrm{j}\mp 1/2}(kr_\mathrm{o}) \\ &\pm H^{(1)}_{\mathrm{j}+1/2}(kr_\mathrm{i})H^{(2)}_{\mathrm{j}\pm 1/2}(kr_\mathrm{o})\},\end{aligned} \tag{8}$$

where $H_\nu^{(K)}(x)$ is the Kth Hankel function and $k = \sqrt{\pi|n|}$ denotes the wave vector.

By using Supplementary Eq. 6 one can generate theoretical curves corresponding to our devices. In Supplementary Fig. 2e, the measured conductance is compared with three theoretical curves and the data are found to agree well when a contact resistance of 420 Ω is taken into account. Note that if we would linearize the dependence of G vs. n using R_c, a procedure which is expected be valid for long-range Coulomb scattering, then the contact resistance would grow from the above value. In our understanding, the estimated R_c is mostly coming from the central leads where the multiple e-beam steps may create the lattice defects. Additionally, in our fabrication process, the development process for central leads has limitation to wash out the residual resists. These resists may forms the dominant charge density under the central metallic leads [5]. Either or both phenomena could explain the origin of this contact resistance of 1 kΩ per micrometer, which is quite close to the experimental findings of Russo *et al.* [6].

Shubnikov-de Haas Oscillations and Quantum Mobility

Samples were characterized at low magnetic fields around a few hundred mT using Shubnikov-de Haas oscillations (the well-defined quantum Hall states in our samples first develop around 0.5 T). These oscillations yield information about the characteristic properties of charge carriers like mobility, quantum life time of carriers, and their effective mass. The diagonal resistance R_xx oscillates periodically with inverse magnetic field, while temperature suppresses these oscillation due to enhanced scattering of carriers. The variation of resistance is given by equation [7]:

$$\frac{R_\mathrm{xx}}{R_0} = 1 + 4\frac{X}{\sinh(X)}\exp\left(-\frac{\pi}{\mu_\mathrm{q}\mathbf{B}}\right)\cos\left(\pi\frac{E_\mathrm{F}}{\hbar\omega_\mathrm{c}} - \theta\right), \tag{9}$$

where R_0 is the resistance at zero field, $X(T) = 2\pi^2 k_\mathrm{B}T/\hbar\omega_\mathrm{c}$ with cyclotron frequency $\omega_\mathrm{c} = e\mathbf{B}/m^*$, E_F specifies the Fermi level, μ_q denotes the quantum mobility

of charge carriers, and θ is a phase factor. Looking at resistance oscillations alone, Supplementary Eq. 9 can be written as:

$$\Delta R_{\mathrm{xx}} = A \cdot \exp\left(-\frac{\pi}{\mu_{\mathrm{q}}\mathbf{B}}\right) \cdot \cos\left(\pi \cdot \frac{E_{\mathrm{F}}}{\hbar\omega_{\mathrm{c}}} - \theta\right), \quad (10)$$

with A as a constant defined by $X(T)$.

Supplementary Fig. 3a displays the SdH oscillations seen in sample EV. The quantum mobility obtained from the fit amounts to $\mu_{\mathrm{q}} \simeq 6 \times 10^4$ cm^2/Vs, which is by a factor of two smaller than the value of the field effect mobility in Table I of the main paper. This value for μ_{q} corresponds to the quantum scattering time $\tau_{\mathrm{d}} \simeq 0.3$ ps which yields for the Landau level width $\delta_{\mathrm{LL}} = \hbar/2\tau_{\mathrm{q}} \simeq 10$ K. In order to achieve a better analysis of the oscillations themselves, a base line marked by the red line in Supplementary Fig. 3a was subtracted from R. These pure oscillations are displayed in Supplementary Fig. 3b. Quantum mobility was extracted from the envelope curve of these oscillations, as indicated in Supplementary Fig. 3b. In the case of sample EV, the quantum mobility is $\mu_{\mathrm{q}} = 6 \times 10^4$ cm^2/Vs (red lines in Supplementary Fig. 3b).

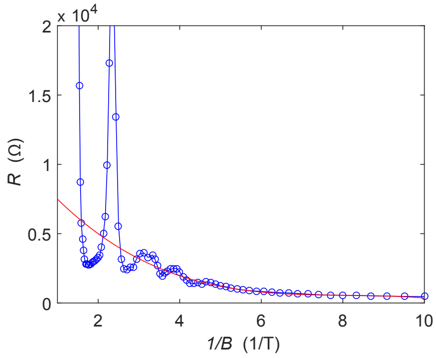

(a) Shubnikov-de Haas Oscillations

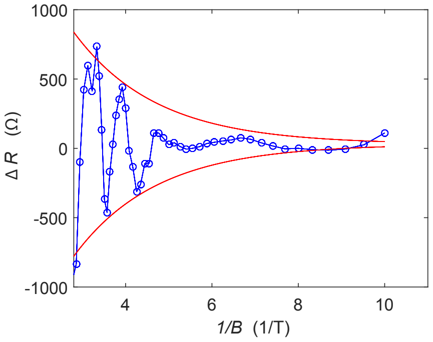

(b) Quantum mobility

Supplementary Figure 3: Quantum mobility calculation: **a)** Resistance of sample EV as a function of the inverse of the perpendicular magnetic field $1/\mathbf{B}$ at V_{g} = 10 V. Shubnikov-de Haas oscillations are visible around the base line indicated by the red curve. **b)** Shubnikov-de Haas oscillations around the subtracted base line (red curve in Supplementary Fig. 3a). The solid red curves represent envelope of the oscillations and they correspond to quantum mobility $\mu_{\mathrm{q}} = 6 \times 10^4$ cm^2/Vs.

Supplementary Note 4: Analysis of Transconductance Lines

The transconductance g_{m} measurements were performed to identify fragile fractional fractional quantum Hall states in a well-controlled, indisputable manner. For $g_{\mathrm{m}}(n,\mathbf{B})$ measurement, an ac excitation of the 2.7 $\mathrm{mV_{rms}}$ is applied to the back gate on top of the dc voltage, and the sample is voltage biased with $V \sim 0.1-3$ mV. Both amplitude and phase of the current modulation were recorded using a lock-in amplifier. The gate modulation frequency was selected to correspond to the roll-off frequency of RC time constant, governed by the sample resistance of a few MΩ. Consequently, the change in g_{m} was observed both in the phase angle and amplitude of the recorded signal. It turned out that, when using long integration times in our lock-in detection, the phase signal was easier to analyze for identification of FQH states than the amplitude signal. Hence, we found our analysis mostly on the phase of the transconductance signal.

Transconductance-based Landau fan type of diagram for sample XD is displayed in Supplementary Fig. 4b. Compared with Fig. 2**b** in the main paper, this scan has more overlapping fringes, which tends to broaden the observed features and to smear the results of the correlation analysis. The number of visible features indicates a stronger disorder potential present in this device compared with sample EV, which is in agreement with the worse field-effect mobility listed in Table I for sample XD.

The FQH state signatures in g_{m} are identified using correlation analysis, which is based on the work of Lee *et al.* [8]. A line with a slope corresponding to a filling factor ν, is drawn on the pixel map of $g_{\mathrm{m}}(n,\mathbf{B})$ and the nearest pixels $(n_{\mathrm{i}},\mathbf{B}_{\mathrm{j}})$ are selected. The values at selected points $(n_{\mathrm{i}},\mathbf{B}_{\mathrm{j}})$ are transferred to vectors $A_{\mathrm{k}}^{\mathrm{w}}$ where the index k covers the full line and the index w keeps track of the vertical ($\mathbf{B}$) coordinate of the drawn lines (see Supplementary Fig. 4a). The cross-correlation $h(\nu)^{\mathrm{w}}$ of pixels within a single vector $A_{\mathrm{k}}^{\mathrm{w}}$ is calculated using

$$h(\nu)^{\mathrm{w}} = \frac{1}{S}\sum_{\mathrm{kl}} A_{\mathrm{k}}^{\mathrm{w}} \cdot A_{\mathrm{l}}^{\mathrm{w}}(1-\delta_{\mathrm{kl}}), \tag{11}$$

where the Kronecker delta δ_{kl} removes the auto-correlation part from the function and S is the total number of pixel products in the summation over k and l.

The total unscaled correlation function is obtained by summing over all values of w: $h(\nu)=\sum_{\mathrm{w}} h(\nu)^{\mathrm{w}}$. In order to find dominant fractional slopes in the data, the cross-correlation was calculated for all $\nu \in \{0...1.5\}$ and histograms of $h(\nu)$ were plotted. A peak in the correlation histogram indicates presence of a dominating feature corresponding to the filling factor ν. For a better inter-comparison of different analyzed regions, we scale our correlation histogram by the maximum correlation: $c(\nu)=h(\nu)/h(\nu)_{\max}$.

The correlation map for sample XD is shown in Supplementary Fig. 4c. As already mentioned above, the correlation peaks in this picture are broadened in comparison with Fig. 2**c** of the main paper. This is because the disorder potential in sample XD is larger than in EV, and disorder will in general suppress the observation of FQH states via life time broadening. Nevertheless, the transconductance measurement is able to bring about many of the fractional states in spite of the disorder.

The data in Fig. 2**b** in the main text display also clear fringes with slopes $\nu = 1$ and 2 overlaid in the middle of the picture on the bulk fringes. This coexistence of fringes representing different filling factors ν requires the local charge density $n_{\mathrm{loc}} > e\mathbf{B}/h$. Furthermore, these slopes are by far off from the possible slopes facilitated by the width of the residual charge distribution based on $\log G(V_{\mathrm{g}})$ analysis. Hence, we conclude that $\nu = 1$ and 2 fringes are located at the boundary regions near the metallic contacts. Due to absence of any polarizing medium close to suspended graphene, the screening of charge near metallic contacts is rather weak. Consequently, strong doping from a metallic contact can extend appreciably in to the suspended graphene near the contact [9]. The amount of edge doping depends on the details of the metallic contacts, which may be in the range of $E_{\mathrm{F}} \sim -100$ meV [4]. Such a doping corresponds to a charge density of $n_{\mathrm{c}} = 7.2 \times 10^{11}$ cm$^{-2} > e\mathbf{B}/h$ under the contact metal, which is sufficient for accommodating QH states with $\nu = 2$.

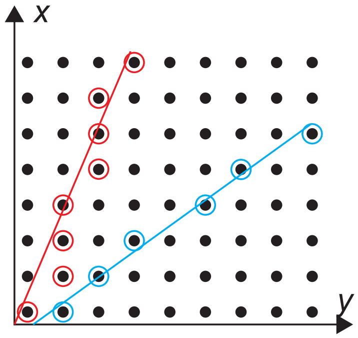

(a) Transconductance analysis scheme

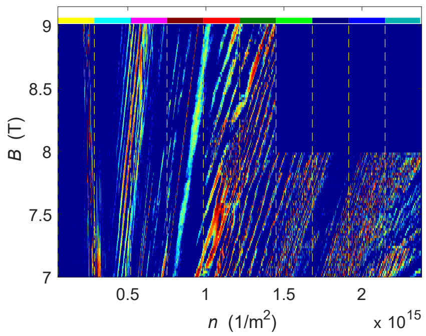

(b) Transconductance for XD

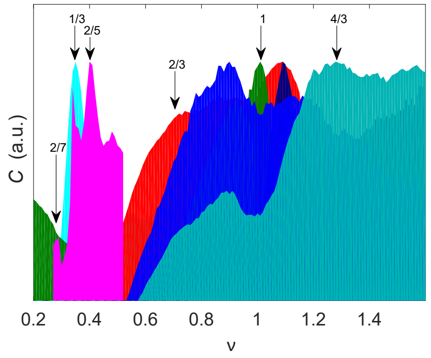

(c) Fractional states identification

Supplementary Figure 4: Transconductance analysis: **a)** Vector $A_{\mathrm{k}}^{\mathrm{w}}$ with slope ν is formed by selecting a line $\mathrm{y} = \nu\mathrm{x} + \mathrm{w}$ on the measured $n - \mathbf{B}$ plane and taking the pixels closest to the line on each row (*i.e.* a particular field $\mathbf{B}$) as illustrated in the figure. Blue and red lines correspond to filling factor slopes ν_1 and ν_2, respectively, and the black dots denote data pixels: the selected data points for correlation analysis are indicated by blue and red circles for these two filling factors. **b)** Measured transconductance phase data at high field $7 - 9$ T for sample XD (the blank upper right corner was not scanned in the experiment). The dashed yellow lines mark the boundaries of the sections in which the correlation function was evaluated. The color on top of each stripe corresponds to the analyzed histogram in Supplementary Fig. 4c. **c)** Scaled correlation function $c(\nu)$ obtained from the data in Supplementary Fig. 4b. The identified fractional states are marked by arrows; states marked in red could not be resolved in conductance measurements directly.

Supplementary Note 5: Magnetoconductance

The Landau fan diagram measured on sample EV (see Table I in the main paper) is illustrated in Supplementary Fig. 5a. In addition to several basic integer Landau levels (2, 6, 10, 14) and broken symmetry states (1, 3, 4), several fractional states such as 1/3, 2/5, 3/7, 4/7, 3/5, and 2/3 are visible in the conductance. However, the levels are visible on the electron side but not clearly on the hole side [1]. Consequently, our magnetoconductance work concentrates on the FQH states on the electron side.

Supplementary Fig. 5b illustrates the data of Supplementary Fig. 5a after differentiation with respect to charge density. By comparing these two figures for G_d and $d\log(G_\mathrm{d})/dn$, it becomes immediately clear that the differentiated data provides a much better resolution for identifying weak fractional states, including those of interacting composite fermions (CF). This is well evidenced by the data displayed in Fig. 2**a** in the main paper.

The interacting composite fermion states are visible also in our data on the differential inverse magnetoconductance $G_\mathrm{d}^{-1} = dV/dI|_{V=0}$, which is depicted in Fig. 3**a** in the main paper for sample EV2. The data display two interacting CF states at $\nu = 4/11$ and $\nu = 4/13$, located on opposite sides of 1/3 state. The G_d^{-1} data on sample EV2 is replotted in Supplementary Fig. 5c as a surface plot on ν vs. $\mathbf{B}$ plane. We note that, in this plot, all peaks assigned to FQH states are well vertical and they do not shift with field over the range $\mathbf{B} = 6-8.5$ T.

The data in Supplementary Fig. 5c clearly indicates the independence of the peaks at $\nu = 1/3$ and at $\nu = 4/11$: the peak at $\nu = 4/11$ appears narrower and it emerges at clearly different field value as the peak at $\nu = 1/3$. The same conclusions apply for the comparison of $\nu = 1/3$ with the peak at $\nu = 4/13$. The signature at $\nu = 4/11$ was also confirmed in mixing current experiments probing non-linear conductance at higher frequencies around 20 MHz where the hopping conductance becomes stronger.

In addition to sample EV2, the interacting CF states $4/11$ and $4/13$ are well visible in the $G_\mathrm{d} = dI/dV$ vs. ν traces of sample EV measured at magnetic fields $6-7.5$ T. These data are displayed in Supplementary Fig. 5d. In these data, the state $4/11$ is better visible than $4/13$, which is to be expected since the former fraction is related to $2\Phi_0$ composite fermion series while the latter belongs to interacting $4\Phi_0$ CF particles.

[1] Asymmetrical magnetoconductance *i.e.* absence of fractional quantum Hall states on *p* side may be caused by the presence of snake states at the *pn* interface, which makes the nature of the particles at this interface to oscillate between an electron and a hole. Such conversion oscillation, when coupled to localized states of hole carriers, is quite likely to lead to broadening of the localized Landau levels.

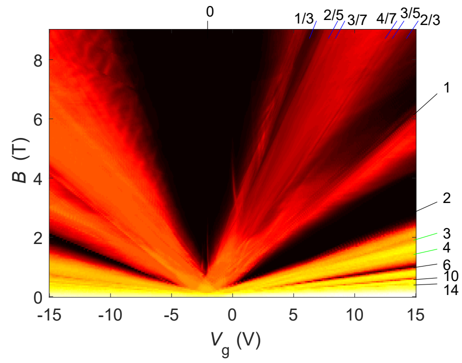

(a) Magnetoconductance $\log(G_{\mathrm{d}})$ Landau fan diagram

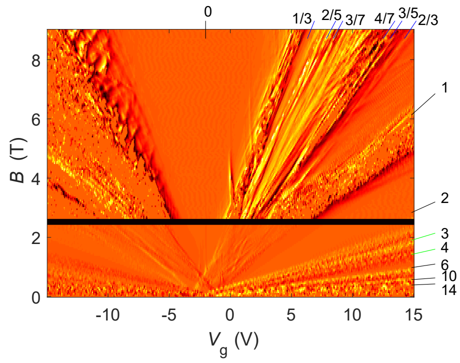

(b) Differentiated magnetocoductance $\delta\log(G_{\mathrm{d}})/\delta n$ Landau fan diagram

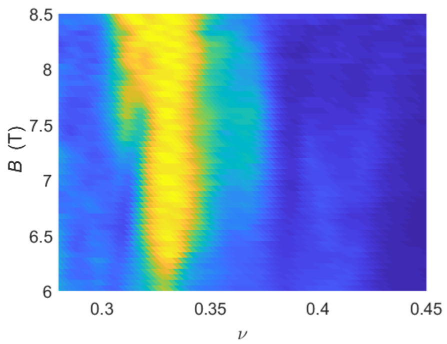

(c) Composite Fractional states identification

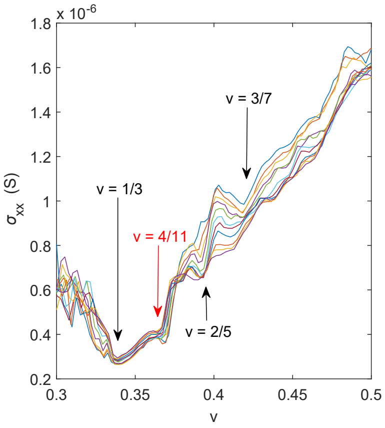

(d) Differential conductivity σ_{xx}

Supplementary Figure 5: Magnetoconductance analysis: **a)** The magnetoconductance ($\log(G_{\mathrm{d}})$) measurement of sample EV showing broken symmetry states $\nu = \{0, 1, 3, 4\}$. Here charge density ranges over $n = -1.1 \times 10^{11} \ldots + 1.5 \times 10^{11}$ cm^{-2} and $G_{\mathrm{d}} = dI/dV|_{V=0}$. **b)** The magnetoconductance ($\log(G_{\mathrm{d}})$) measurement of Supplementary Fig. 5a differentiated with respect to charge density. The high field region, where the scale is by a factor of 100 smaller than the low-**B** region, is separated using a horizontal black stripe. **c)** Inverse magnetoconductance G_{d}^{-1} on the plane spanned by the filling factor ν and magnetic field **B** measured for sample EV2. Interacting CF states $4/13 = 0.308$ and $4/11 = 0.363$ are observed next to $\nu = 1/3$ state above critical field values of 6.8 and 6.3 T, respectively. **d)** The differential conductivity $\sigma_{\mathrm{xx}} = G_{\mathrm{d}} \frac{\ln(r_{\mathrm{o}}/r_{\mathrm{i}})}{2\pi}$ vs. the filling factor ν for sample EV, measured at magnetic fields $\mathbf{B} = 6 - 7.5$ T.

Supplementary Note 6: Basic transport processes

The IV characteristics of our Corbino samples contain contributions from many different transport processes: 1) quantum tunneling, 2) thermally assisted quantum tunneling, 3) thermal activation, 4) phonon assisted hopping, 5) electron crystal sliding *a la* charge density wave. All these processes leave their characteristics in to the IV curves. Furthermore, we have measured IV characteristics under rf-irradiation (see Supplementary Note 9) at which the transport is influenced by photon assisted hopping, 6). Here we will discuss only those topics which are most relevant for the explanation of our data, namely items 3 and 6 above. However, one should keep in mind that processes in items 2 and 3 are intimately connected, and parameters for thermal activation may become adjusted by quantum tunneling near the top of the energy barrier.

Thermal activation

The conductance in the bulk of a quantum Hall conductor depends intimately on the potential landscape of the state of the system. The potential landscape leads to localized states as illustrated in Supplementary Fig. 6a. Theoretical models in the case of a long-range random disorder potential predict thermally activated conductivity,

$$\sigma = A\exp(-U_{\rm s}/2k_{\rm B}T), \tag{12}$$

where the activation energy $U_{\rm s} \simeq C\Delta_\nu$ is proportional to the gap Δ_ν in FQH states with $C \lesssim 1$, and the prefactor $A \simeq (e^*)^2/h$ is governed by effective charge e^* of charge carriers [10, 11]; in thermal activation, the effective charge is related to the filling factor $e^* = \nu e$ [10, 11], whereas in quantum tunneling regime it may be either νe or e [12]. The suppression factor C of Δ_ν in the activation formula depends on the significance of quantum tunneling near the top of the barrier. In a similar approximative approach, the conductivity in the quantum tunneling regime can be expressed as $\sigma = A\exp(-U_{\rm s}/2k_{\rm B}T_{\rm cr})$, where $T_{\rm cr}$ denotes the cross-over temperature between thermal activation and quantum tunneling. The cross-over temperature is difficult to determine theoretically but it can be estimated from the saturation temperature of $\sigma(T)$ scans, which in our experiments varies in the range $T = 1-0.5$ K.

Our conductance at high T agrees with the thermal activation process [10, 11] and, in this regime, the data can be employed to obtain estimates of the excitation energy gaps of the FQH states by assuming $\Delta_\nu = U_{\rm s}$. By determining the activation energy across $\nu = 1/2$ and assuming linear dependence for Δ_ν vs ν, we obtain approximatively 0.2 meV for the composite fermion LL width. This can be regarded as a systematic uncertainty (offset) for the energy gaps quoted in our

paper for FQH states. Supplementary Fig. 6b displays conductance data for the state $\nu = 4/3$ for the sample EV2. The data indicate thermally activated conduction at temperatures above 1 K. By using Supplementary Eq. 12, we obtain for the energy gap $\Delta_{4/3} = 0.7$ K. At low temperatures, especially at low filling factors, the role of thermal activation becomes weaker, and the conductance and *IV* characteristics display a more complex dependence on charge density as will be discussed in Supplementary Note 7.

Photon assisted hopping

Hopping conduction in a disordered conductor having localized states is enhanced by microwave irradiation. This problem has been reviewed e.g. by Efros and Shklovskii [13]. We will deal with this issue only qualitatively, and our discussion is inspired by the review of Galperin and Gurevich [14].

It was first shown in Ref. [15] that, under microwave irradiation, the hopping conduction across localized states is determined by so called close pairs. Each such pair consists of one localized state which is occupied by an electron and one localized state which is empty, the distance between them being much smaller than the average distance between localized states given by $\langle \mathrm{r} \rangle = (\frac{4\pi^2}{3} N_A)^{-1/2}$ where N_A is the density of localized states. Due to absorption, electron occupancies in the pairs are changed, which is equivalent to an enhancement of the average energy of bound electrons. Thus, microwave absorption leads effectively to heating of the system of localized states. Below we determine the conductivity $\sigma(\omega)$ that is relevant for the absorption of irradiation in the presence of localized states in a three dimensional system. As usual in hopping conductance, we don't expect any qualitative change between different dimensionalities, only a change in the exponent α of the conductivity $\sigma \propto \exp(\left(\frac{E_0}{E}\right)^{\alpha})$ in terms of characteristic energy. This difference in the exponent α is irrelevant for the following discussion.

We are interested in the regime in which $e^2/(4\pi\varepsilon\langle \mathrm{r} \rangle) >> \hbar\omega, k_\mathrm{B}T$, i.e. the charging energy of the localized state is the largest energy scale. In the resonant case ($\hbar\omega$ matches the energy difference between the levels in question), the Ohmic microwave conductivity is given by

$$\sigma_0^{\mathrm{res}}(\omega) = \frac{\pi^2}{3} a g^2 \frac{e^4}{\varepsilon} \omega r_\omega^3 \tanh\left(\frac{\hbar\omega}{k_\mathrm{B}T}\right). \qquad (13)$$

Here g denotes the single particle density of states, a corresponds to the radius of the localized state, and

$$r_\omega = a \log\left(\frac{\Lambda_0}{\hbar\omega}\right) \qquad (14)$$

specifies the minimum distance between pairs with energy separation $\hbar\omega$; Λ_0 denotes the off-diagonal elements in the tunnel coupling matrix. As r_ω does not depend on temperature, the resonant conductance $\sigma_0^{\mathrm{res}}(\omega)$ decreases with increasing T. Similarly, a strong drive will result in more equalized populations, which leads to decrease of absorption. In both cases of reduced absorption, relaxation of the close pairs becomes of major importance.

In the non-resonant (relaxational) case with fast relaxation $\omega\tau_1 << 1$, where $1/\tau_1$ describes the relaxation rate due to the environment, the conductance becomes

$$\sigma_0^{\mathrm{rel}}(\omega) \approx ag^2 \frac{e^4}{\varepsilon} \omega r_e^3. \tag{15}$$

In our experiments based on mixing current detection of the FQH states, the relevant frequency is around 20 MHz (see Note 10). We expect that the localized states will remain incoherent in this regime (i.e. relaxation time $\tau_1 < 50$ ns). In this case, the relevant pairs have an energy separation $E \sim k_\mathrm{B}T$ and a life time $\tau_{\mathrm{min}} \sim \omega$, and the characteristic minimum distance between pairs becomes

$$r_\mathrm{e} = a \log\left(\frac{\Lambda_0}{k_\mathrm{B}T\left[\omega\tau_{\mathrm{min}}(T)\right]^{1/2}}\right). \tag{16}$$

By using electron-phonon supercollision scattering rate near the Dirac point [16], we observe that r_e increases with T, which leads to growth of relaxational conductance $\sigma_0^{\mathrm{rel}}(\omega)$ with temperature.

To sum up this section, hopping conductance grows with ω and, in magnetic fields where $\ell_\mathrm{B} \sim \langle \mathrm{r} \rangle$, we may have $\sigma(\omega) >> \sigma(0)$. In addition, $\sigma_0^{\mathrm{res}}(\omega)$ due to resonant conductance is expected to dominated by non-resonant relaxation, which will enhance both as a function of T and microwave power in the limit of $\omega\tau_1 << 1$. This microwave absorption will result in heating of graphene that, in turn, will govern the change in low frequency (or DC) conductance in the measurements on our sample.

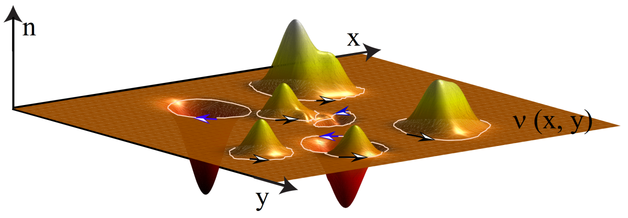

(a) Localized states

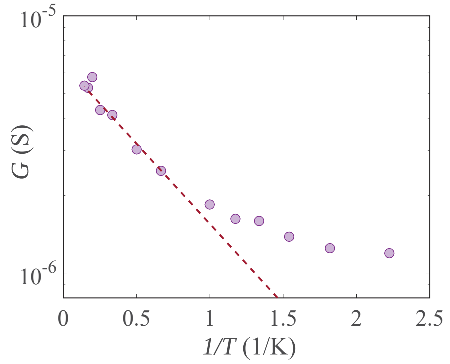

(b) Activation gap energy $\nu = 4/3$

Supplementary Figure 6: Transport process: **a)** Schematic picture of localized states with variation in charge density marked in green (enhanced density) and red (reduced density). The localized states are embedded within the incompressible FQH liquid with constant electron density (brown plane). Landau levels (LLs) are bending upwards when approaching the red regions while they bend down around the green bumps. Consequently, skipping orbits of edge state electrons reside outside and inside the borders of the low and high density regions, respectively. White-blue arrows mark the directions of the currents of these localized edge states. Electric transport at low temperatures takes place via quantum tunneling between the localized states through a barrier due to the energy gap Δ_ν of the incompressible FQH region. In case of considerable variation of electron density (*i.e.* the charge dips/hillocks reaching the next FQH charge density plane), there will be additional localized fractional states within such regions. Since these states are localized within a bounded region with an edge state, their effect on charge transport can be neglected. **b)** Logarithm of the zero-bias conductance G vs. inverse temperature $1/T$ for sample EV2 at magnetic field $\mathbf{B} = 4$ T. The dashed line corresponds to an energy gap of $\Delta_{4/3} = 0.7$ K

Supplementary Note 7: *IV*-characteristics

Our *IV* characteristics at low filling factor ($\nu = 0.16...0.33$) are illustrated in Supplementary Fig. 7a for sample EV. At $\nu = 0.33$, the *IV* curve displays first a linear part that extends nearly up to $0.5 - 0.7$ mV, above which there is a strong increase in current in a power-law-like fashion as V^{α}. Such an *IV* curve can be understood by transport via quantum tunneling along a percolating path of several localized states where the non-linear regime would be a sign of competing order of correlated tunneling of chiral edge states [17, 18].

At higher temperatures, this conductance at $\nu = 0.33$ becomes weakly temperature dependent, which is an indication of thermally assisted quantum tunneling. The combination of thermal activation and quantum tunneling leads to a lowered effective gap as determined from the temperature dependence of conductance [11]. At $V > V_{\mathrm{T}} \simeq 1$ meV, the *IV* characteristics becomes a power law V^{α} with an exponent of $\alpha = 3 - 4$. We assign this to edge-to-edge tunneling of fractional charge between localized states along the percolating path[2].

At filling factor $\nu = 0.20$, even the low voltage part appears as non-linear and it is difficult to resolve any Ohmic part in the *IV* curve. This behavior is identified as a cross-over regime between FQH and Wigner crystal order: Here pinning of the crystallites in the Wigner phase is not fully developed and linear quantum tunneling and non-linear sliding processes of charge are competing, resulting in an *IV* curve that is difficult to classify.

The behavior of our non-linear *IV* characteristics for sample XD is illustrated in Fig. 4**b** in the main paper: the data at $\nu = 0.16$ are seen to display exponential increase in current between $V \sim 0.1 - 2.0$ mV. Here we display the *IV* characteristics of sample EV2 in Supplementary Fig. 7b at filling factor $\nu = 0.15$. The data indicate thermal depinning of Wigner crystal which is non-linear to the lowest detectable currents. Eq. 1 of the main paper has been used to fit the data using parameters $e^* = e$, $T = 0.2$ K, $\Delta = 170$ μV, and $N = 5$. These values are consistent with results on the XD sample as well as with the results of the same sample under rf irradiation (see below).

Supplementary Fig. 7c displays the logarithm of total conductance $G_{\mathrm{tot}} = I/V$ on the $\nu - V$ plane for sample XD. The linear low-bias structure of the *IV* curves at $\nu > 0.2$ is seen as constant conductance regions below the power-law regime. Above the power-law regime, G_{tot} gradually approaches the high-bias slope $dI/dV \sim$ 100 kΩ^{-1}. Supplementary Fig. 7c indicates that conductance is strongly suppressed between $\nu = 0.12 - 0.20$, but good conductance re-emerges again at $\nu =$

[2]Our exponent for tunneling between localized states above thermal energy is approximately twice that observed for metal-to-edge tunneling, see Jolad[19]

$0.10-0.12$. For sample EV2, the Wigner crystal state found over a slightly smaller regime covering $\nu = 0.15-0.18$.

The *IV* characteristics of EV2 in the re-entrant good conductance regime at filling factor $\nu = 0.14$ is illustrated in Supplementary Fig. 7d. The behavior at $\nu = 0.14$ resembles that of *IV* curves at $\nu = 1/3$, where the initial linear behavior changes to a power law with an exponent $\alpha = 3-4$, but now with an exponent $\alpha \simeq 7$. The shape of the *IV* curve hints towards re-entrance of FQH states, but we are not able to identify any peak in the gate sweeps, which would make this conjecture more plausible. Nevertheless, as displayed in Fig. 3B in the main paper, we have determined the energy gap using $G(T)$ measurements and we can state that this intermediate conductance state is gapped. At smaller charge density, for example at $\nu = 0.12$, the *IV* curve displays a larger gap than in the Wigner crystal regime, and the behavior gradually develops towards Zener tunneling *IV* curves.

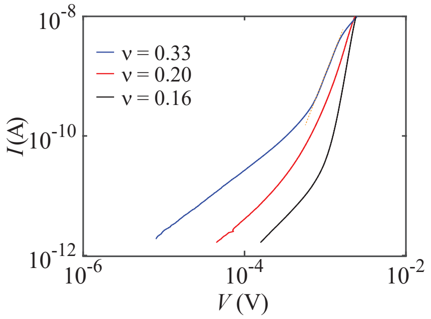

(a) *IV* curves at low charge densities

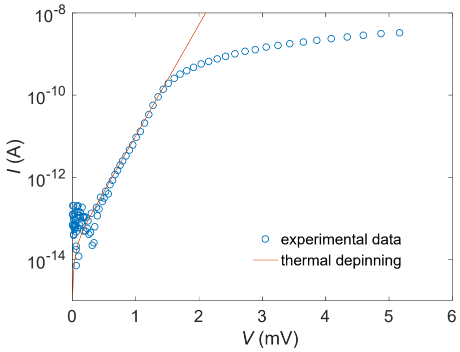

(b) *IV* characteristics of Wigner crystal

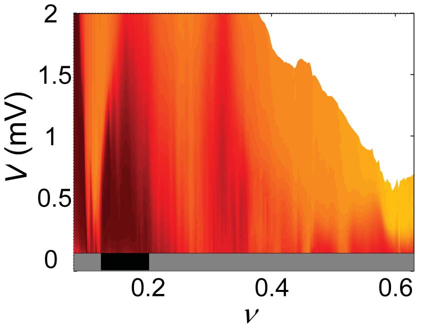

(c) Density plot of *IV*'s

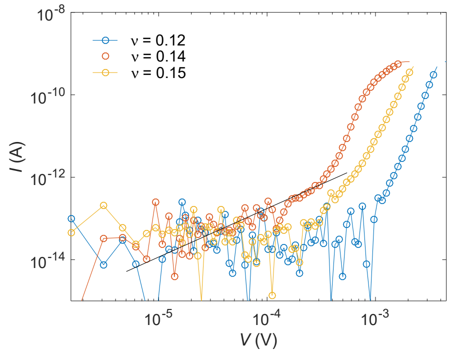

(d) *IV* analysis of re-entrant Fermi liquid

Supplementary Figure 7: *IV* characterization **a)** *IV* curves on log-log scale measured for sample EV at $\mathbf{B} = 9$ T for filling factors $\nu = 0.33$ (blue), 0.20 (red), and 0.16 (black). The fitted dashed line to $\nu = 0.33$ data in the non-linear regime indicates a power law with an exponent of $\alpha \sim 3.4$. **b)** *IV* curve of sample EV2 on semi-log scale measured at $\nu = 0.15$. The solid line indicates the exponential growth obtained from the thermal depinning model (Eq. 1 in the main paper) using parameters $e^* = e$, $T = 0.2$ K, $\Delta = 170$ μV, and $N = 5$. **c)** Logarithm of conductance $G_{tot} = I/V$ measured for sample XD as a function of bias voltage $V = 0 \ldots 2$ mV over filling factors $\nu = 0.05 - 0.65$ at $\mathbf{B} = 9$ T; orange color indicates high conductance and dark red shows reduced conductance (range spans $2 \cdot 10^{-5} - 1 \cdot 10^{-8}$ S). The Wigner crystal regime $\nu = 0.12 - 0.20$ is indicated by the black bar on the grey bottom stripe. **d)** *IV* curves of sample EV2 measured at slightly smaller electron density of the Wigner crystal behavior at $\nu = 0.15$. The solid black line denotes linear *IV* characteristics.

Supplementary Note 8: Resonance modes in pinned Wigner crystal

The nature of pinning in disorder Wigner crystal depends strongly on the characteristics of the disorder. In suspended graphene membrane there are two quite different disorder elements present. First, there are membrane ripples (dimples), which can be either static or alternatively dynamic due to thermal fluctuations. Second, residual charge density is related to charged impurity disorder, most likely caused by residues of the lithography process, which are present in spite of the current cleaning. Static ripples can be modeled by the interfacial disorder model of Ref. [20], which relates the pinning potential of the Wigner crystal to the depth and width of the interfacial dimples. The pinning potential caused by charged impurities in clean graphene has a rather long-scale variation so that the particle size (magnetic length l_B) is smaller than the correlation length of the disorder ξ. Such a pinning case has been treated for example in Ref. [21]. We expect that both pinning mechanisms will contribute to the pinning potential and contribute to the pinning frequency. By assuming that the disorder contributions are independent, we obtain the pinning frequency ω_p as a combination of these two processes: $\omega_\mathrm{p}^2 = \omega_\mathrm{q}^2 + \omega_\mathrm{r}^2$ where ω_q and ω_r refer to charged impurities and interfacial disorder, respectively. Below we will outline the basic pinning energy considerations in both disorder cases.

Pinning by charged impurities

A Wigner solid is pinned in the presence of disorder, which results in breaking of the long-range order of the crystal. Consequently, the crystal splits into coherent, independent crystallites, and a gap opens up in the transverse phonon modes of the Wigner lattice. In our graphene case, the correlation length $\xi > l_\mathrm{B}$, and the particle locally sees a smooth potential, for which the particle size l_B is not directly relevant. Under these conditions, the crystallite size L_c over which collective pinning takes place is independent of the magnetic field. The crystallite size specifies the collective-pinning-mode frequency of the Wigner solid. One obtains for the pinning frequency in the simplest approximation

$$\omega_\mathrm{p}^0(\mathbf{B}) = (2\pi^2)^{-\frac{1}{6}} \frac{cR_\mathrm{a}^{-2}}{n\mathbf{B}} \left(\frac{a}{\xi}\right)^6, \tag{17}$$

where c is the shear modulus of the crystal, a is the lattice constant of the Wigner crystal, and $R_\mathrm{a} = ca^2/n_0\Delta$ is the length scale at which the fluctuation of the electrons in the Wigner crystal starts to be on the order of a; n_0 denotes the average

charge density. Consequently, these kinds of pinning sites yield a contribution to pinning frequency which decreases as $\mathbf{B}^{-1}$. Using realistic values for Δ and ξ, we obtain pinning frequencies in the GHz range. The relation of the pinning frequency f_p to the crystallite size is given by $L_c = (2\pi c/n_\mathrm{i} e \mathbf{B} f_p)^{1/2}$. By assuming the classical form for the shear modulus in a 2D solid, $c = 0.245 e^2 n_\mathrm{i}^{3/2}/4\pi\varepsilon_0\varepsilon_\mathrm{g}$, and using $\varepsilon_\mathrm{g} \sim 3$, we obtain for the domain size of the Wigner crystal L_c = 0.70 μm at $\mathbf{B} = 9$ T. This is approximately half of our sample disk width $r_\mathrm{o} - r_\mathrm{i}$ = 1.15 μm.

Commonly, charged impurities are viewed as a paradigm for a strongly pinned system [22], where certain locations of the lattice are basically fixed and, as such, eliminated as degrees of freedom. However, it is argued in Ref. [20] that one may consider a strongly pinned Wigner crystal as a weakly pinned crystal with point defects. If so, then the strongly pinned Wigner crystal for the purposes of AC conductance is equivalent to a weakly pinned, defective WC. Such arguments provide further support for the general applicability of the weak pinning formulas used in the main paper.

Pinning by ripples of membrane

Here we assume that the dimple lattice of suspended graphene can be approximated by the model of interfacial pits of depth Δz and width w developed in Ref. [20]. The scanning tunneling microscope results of Ref. [23] indicate, for a typical current-cleaned graphene sample such as ours, an amplitude of corrugation of $Z = 0.4$ nm with a radius of curvature $R = 1.5$ nm (i.e. wave length ~ 5 nm). Hence, we may assume $\Delta z = 0.4$ nm and $w = 3$ nm. In our sample, the ratio of $\Delta z/\varepsilon$ is the nearly the same as that used in the evaluations in Ref. [20] (both $\varepsilon_\mathrm{g} = 3$ and $\Delta z \sim 0.4$ nm are by a factor of ~ 3 smaller in graphene when compared with GaAs heterostructures). The potential energy gained by placing an electron into a dimple that is much larger than the magnetic length is given by $\Delta E = 2\pi n_0 e^2 \Delta z/(4\pi\varepsilon_0\varepsilon_\mathrm{g}) \approx 7$ K in temperature units. Since a typical dimple is generally be much smaller than the magnetic length, the pinning energy of an electron trapped in a single dimple will be on order of $\Delta E w^2/l_\mathrm{B}^2 \ll \Delta E$.

As in Ref. [20], we will assume the average distance between dimples $1/\sqrt{n_\mathrm{i}}$ is smaller than the lattice spacing of electrons a but larger than the magnetic length l_B. This means that each unit cell of the Wigner crystal contains several dimples on to which an electron could be trapped. We will ignore the spread in the size distribution of dimples and assume that their distribution can be approximated by single size and uniform density ($> 10^{11}$ cm^{-2}), the latter of which will form a fitting parameter, to be determined self-consistently in the model.

The average number of dimples occupied by an electron in a single domain of size L_c if placed at a random location is given by $N_\mathrm{pin} = \pi n_0 L_\mathrm{c}^2 l_\mathrm{B}^2 n_\mathrm{i}$. Consequently,

the energy per electron gained from the disorder potential $u_{\rm p}$ is

$$u_{\rm p} \approx \Delta E \frac{w^2}{l_{\rm B} L_{\rm c}} \left[\frac{\pi n_{\rm i}}{n_0}\right]^{1/2}. \tag{18}$$

By assuming purely transverse distortions in the lattice, we obtain an estimate for the distortion energy per electron

$$u_{\rm d} \approx \frac{c}{n_{\rm i} L_{\rm c}^2 n_0}$$

where c is the shear modulus of the lattice. Minimizing the total energy $u_{\rm d} - u_{\rm p}$ with respect to $L_{\rm c}$, we obtain for the optimum domain size

$$L_{\rm c} = \frac{2 c l_B}{\Delta E w^2 (\pi n_{\rm i}^3 n_0)^{1/2}}. \tag{19}$$

The resulting pinning energy per particle is found by substituting this value of $L_{\rm c}$ into our expression for $u_{\rm p}$ in Supplementary Eq. 18. Using standard lattice parameters for the shear modulus, the resulting pinning energy per particle can be written as

$$u_{\rm p} \approx 5.24 \frac{4\pi\varepsilon a^3 \Delta E^2 w^4 n_{\rm i}^2}{e^2 l_{\rm B}^2}. \tag{20}$$

In the simplest approximation, the pinning frequency is given by the average binding energy per site, *i.e.*, $\hbar\omega_{\rm pin} \approx u_{\rm p}$. Note that within this approximation, $\omega_{\rm pin} \propto l_{\rm B}^{-2} \propto \mathbf{B}$, which leads to a pinning frequency that *increases* with magnetic field. Using $n_{\rm i} \approx 2.5 \times 10^{11}$ cm^{-2}, $l_{\rm B} \approx 9$ nm, $w = 3$ nm, and $\Delta E / k_{\rm B} = 7$ K, the pinning frequency becomes ~ 1.5 GHz. This value is strongly dependent on ΔE or the dimple size w, which could even be larger than 3 nm [23].

To sum up this section, we have two independent pinning potentials for the electrons in the Wigner phase. Charged impurities lead to pinning frequency $\omega_{\rm q} \propto \mathbf{B}^{-1}$ while dimples yield $\omega_{\rm r} \propto \mathbf{B}$. These pinning frequencies are added as independent contributions so that they yield the total pinning frequency $\omega_{\rm p}^2 = \omega_{\rm q}^2 + \omega_{\rm r}^2$. Since the pinning frequency does not display much magnetic field dependence, $|df_{\rm p}/d\mathbf{B}| < 100$ MHz/T, we conclude that both contributions are approximately of equal magnitude. Consequently, the pinning frequency used in deducing the crystallite size, is only approximative and it yields an upper limit for the crystallite size.

Supplementary Note 9: Wigner crystal under rf irradiation

Commonly, microwave conductance measurements have been conducted on GaAs 2DEG by capacitively coupling RF power to the 2DEG and measuring its transmission through the sample (at high frequency). FQHSs, Wigner phases, and CDW-phases have been studied with this technique [24, 25, 26, 27, 28]. Similar to earlier works, we have also probed the Wigner crystal by microwave spectroscopy. Our measurement method has two distinct features; first, the rf power is directly coupled to the Wigner crystal via the inner contact of the Corbino geometry and, second, the transport across the Wigner crystal is measured at DC. Irrespective of these differences compared with earlier studies in 2DEG, the essence of our incompressibility measurements of the Wigner crystal under rf radiation will be the same.

A schematic of the measurement circuit is depicted in Supplementary Fig. 8. We employed bias-T circuits (Minicircuits) at the mixing chamber plate in order to apply simultaneously DC and AC bias across the sample. DC (or low-frequency) conductance was measured in voltage bias configuration through inductors while microwaves were coupled via capacitors to the sample. In these conductance measurements, a DC bias of $V = 50\ \mu V$ was applied to the inner contact while the resulting current at the outer contact was tracked using a Stanford SR570 current (transimpedance) preamplifier operating at a gain of 10^{10} V/A followed by a HP 34410A multimeter. The DC bias was provided using HP 33120A generator connected through a 1:2000 voltage divider.

Anritsu MG3692B rf-generator was used to generate the applied rf irradiation at frequencies f = 10 MHz - 10 GHz. The rf-input line had an attenuation of 50 dB at f = 1 GHz, with additional $\sim$10 dB per decade in frequency (i.e. attenuation was roughly 40 dB at 100 MHz, and 60 dB at 10 GHz). The rf-signal was fed to the inner contact through the bias-T, while the rf-line was terminated using a 50 Ω resistor at the outer contact.

There should always be hopping conductance of Efros-Shklovskii type, even in the case of a disordered Wigner crystal [29]. As discussed in some detail in Supplemetary Note 6, this conduction is mediated by resonant pairs of localized states, the absorption of which grows with enhanced frequency. Since there is a large spread of energy differences among the resonant pairs in our sample, we expect to have an increase of absorption over a large band of irradiation frequencies. This is apparent in our experiments at large irradiation power as displayed in Supplementary Fig. 9 for the strongly insulating regime at the filling factor $\nu = 0.12$. The rf absorption leads to increase of conductance via heating, and this enhancement in G is seen to increase monotonically with frequency in Supplementary Fig. 9.

At high irradiation power, Joule heating $P = \sigma(\omega) v_{\rm ac}^2$ becomes relevant, and

it leads to an increase of temperature and conductance in the sample. At low irradiation powers (around $-70\ldots-65$ dBm), however, only one strong peak in DC conductance around 3 GHz is observed (see Fig. 4**a** in the main paper). The resonant frequency coincides well with estimates for a resonance in a pinned, disordered Wigner crystal.

The amplitude of this conductance resonance with respect to a broad background current enhancement grows with temperature. We assign this behaviour to a combined action of activated conductance and enhanced Joule heating by the microwave irradiation in the Wigner crystal. The Joule heating $P = \sigma(\omega)v_{\mathrm{ac}}^2$ of the electron system is strongly frequency dependent due to the resonant behavior of $\sigma(\omega)$ at pinning frequency ω_{p}. At the resonance, increase in temperature leads to an increase in observed conductance, and the change in conductance per unit Kelvin (ΔT) will grow with increasing temperature due to increased microwave absorption. The conductance enhancement will continue till the Wigner crystal melts and the resonance absorption at $\sigma(\omega_{\mathrm{p}})$ is lost. This scenario supports the identification of the maximum in Fig. 4**c** of the main paper as the melting temperature of the Wigner crystal. Similar identification of the Wigner crystal melting has been employed in Ref. [30]. For reference, we also show $G(T)$ without rf irradiation in Supplementary Fig. 10. This plot on linear scale indicates beginning of melting at $T_{\mathrm{mb}} = 1.4$ K, which is lower but consistent with the melting temperature $T_{\mathrm{m}} = 1.7$ K obtained from rf resonance measurements.

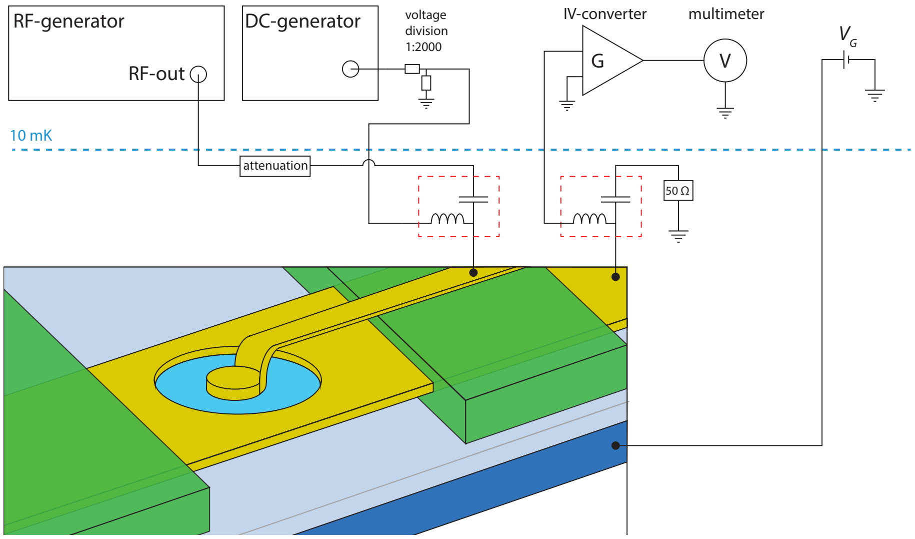

Supplementary Figure 8: rf measurement setup: Schematic of the setup for conductance measurements under rf irradiation. Cryostat details and low pass filters in the DC-lines have been omitted from the picture for clarity.

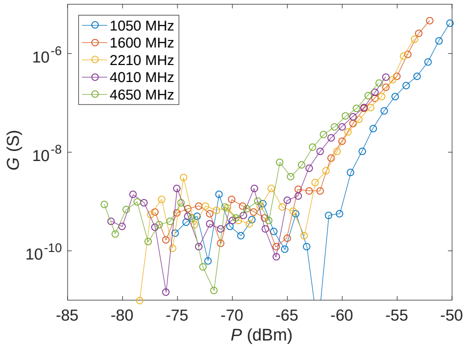

Supplementary Figure 9: rf conductance analysis: Conductance as a function of input rf power at the Corbino disk measured in the frequency range $1-5$ GHz for EV2 at $\nu = 0.12$ (strongly gapped regime). The applied DC-bias was $V = 50$ μV. A clear increase in the DC conductance $G = I/V$ is observed at power levels exceeding ~ -65 dBm. The power level used in the measurement of Fig. 4**a** in the main paper corresponds to -68 dBm in this graph.

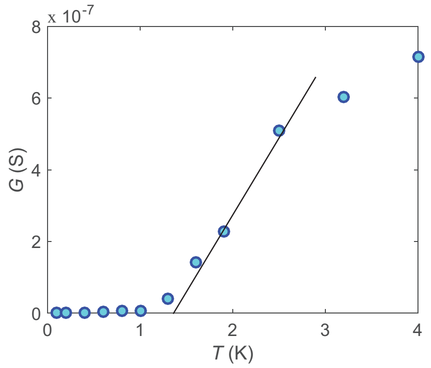

Supplementary Figure 10: Melting of Wigner crystal: Temperature dependence of conductance $G(T)$ for sample EV2 on linear scale at measured at $\nu = 0.15$. The line marks linear growth, using which one obtains approximate melting temperature of $T_{\mathrm{mb}} = 1.4$ K for the Wigner crystal phase.

Supplementary Note 10: FQH states under rf irradiation

In addition of studies of the FQH states under GHz irradiation, we have studied them at MHz frequencies using mixing currents facilitated by mechanical motion[31]. The mechanical motion with amplitude z yields a mixing current via differential conductance $G_\mathrm{d}(\omega)$ as

$$I_\mathrm{mix} \propto \frac{\partial G_\mathrm{d}(\omega)}{\partial z}\left|\frac{\partial \mathrm{Re}(z)}{\partial f}\right|$$

when the sample is biased using a frequency modulated signal $V^\mathrm{FM}(t) = V^\mathrm{AC}(\cos(2\pi f t + (f_\mathrm{A}/f_\mathrm{L})\sin(2\pi f_\mathrm{L} t))$. Here V^AC is the amplitude of the carrier frequency signal, f_L is the frequency modulation, and f_A defines the modulation depth. In the Wigner crystal and FQH transport regimes, the hopping conduction is enhanced by operation frequency so that the mixing current method may be more sensitive than the low frequency or DC conductance measurement. We have carefully measured the region around $\nu = 1/3$ and our experiments confirm the finding of the fraction state of $\nu = 4/11$ observed in the low frequency measurements.

References

[1] Huang, Y. *et al.* Reliable exfoliation of large-area high-quality flakes of graphene and other two-dimensional materials. *ACS Nano* **9**, 10612–10620 (2015).

[2] Tombros, N. *et al.* Large yield production of high mobility freely suspended graphene electronic devices on a polydimethylglutarimide based organic polymer. *J. Appl. Phys.* **109**, 093702 (2011).

[3] Rycerz, A., Recher, P. & Wimmer, M. Conformal mapping and shot noise in graphene. *Phys. Rev. B* **80**, 125417 (2009).

[4] Laitinen, A. *et al.* Contact doping, Klein tunneling, and asymmetry of shot noise in suspended graphene. *Phys. Rev. B* **93**, 115413 (2016).

[5] Huard, B., Stander, N., Sulpizio, J. A. & Goldhaber-Gordon, D. Evidence of the role of contacts on the observed electron-hole asymmetry in graphene. *Phys. Rev. B* **78**, 121402 (2008).

[6] Russo, S., Craciun, M., Yamamoto, M., Morpurgo, A. & Tarucha, S. Contact resistance in graphene-based devices. *Physica E: Low-dimensional Systems and Nanostructures* **42**, 677 – 679 (2010).

[7] Ando, T., Fowler, A. B. & Stern, F. Electronic properties of two-dimensional systems. *Rev. Mod. Phys.* **54**, 437–672 (1982).

[8] Lee, D. S., Skákalová, V., Weitz, R. T., von Klitzing, K. & Smet, J. H. Transconductance fluctuations as a probe for interaction-induced quantum Hall states in graphene. *Phys. Rev. Lett.* **109**, 056602 (2012).

[9] Khomyakov, P. A., Starikov, A. A., Brocks, G. & Kelly, P. J. Nonlinear screening of charges induced in graphene by metal contacts. *Phys. Rev. B* **82**, 115437 (2010).

[10] Polyakov, D. G. & Shklovskii, B. I. Universal prefactor of activated conductivity in the quantum Hall effect. *Phys. Rev. Lett.* **74**, 150–153 (1995).

[11] d'Ambrumenil, N., Halperin, B. I. & Morf, R. H. Model for dissipative conductance in fractional quantum Hall states. *Phys. Rev. Lett.* **106**, 126804 (2011).

[12] Hashisaka, M., Ota, T., Muraki, K. & Fujisawa, T. Shot-noise evidence of fractional quasiparticle creation in a local fractional quantum Hall state. *Phys. Rev. Lett.* **114**, 056802 (2015).

[13] Efros, A. L. & Shklovskii, B. *Electron-electron interaction in disordered systems* (North-Holland: Amsterdam, 1985).

[14] Galperin, Y. M. & Gurevich, V. L. *Hopping Transport in Solids* (Elsevier Science Publishers, 1991).

[15] Pollak, M. & Geballe, T. H. Low-frequency conductivity due to hopping processes in silicon. *Phys. Rev.* **122**, 1742 (1961).

[16] Laitinen, A. *et al.* Electron - phonon coupling in suspended graphene: Supercollisions by ripples. *Nano Letters* **14**, 3009–3013 (2014).

[17] Chang, A. M., Pfeiffer, L. N. & West, K. W. Observation of chiral Luttinger behavior in electron tunneling into fractional quantum Hall edges. *Phys. Rev. Lett.* **77**, 2538–2541 (1996).

[18] Grayson, M., Tsui, D. C., Pfeiffer, L. N., West, K. W. & Chang, A. M. Continuum of chiral Luttinger liquids at the fractional quantum Hall edge. *Phys. Rev. Lett.* **80**, 1062–1065 (1998).

[19] Jolad, S., Sen, D. & Jain, J. K. Fractional quantum Hall edge: Effect of nonlinear dispersion and edge roton. *Phys. Rev. B* **82**, 075315 (2010).

[20] Fertig, H. Electromagnetic response of a pinned Wigner crystal. *Phys. Rev. B* **59**, 2120–2141 (1999).

[21] Chitra, R., Giamarchi, T. & Le Doussal, P. Pinned Wigner crystals. *Phys. Rev. B* **65**, 035312 (2001).

[22] Fukuyama, H. & Lee, P. A. Pinning and conductivity of two-dimensional charge-density waves in magnetic fields. *Phys. Rev. B* **18**, 6245–6252 (1978).

[23] Zan, R. *et al.* Scanning tunnelling microscopy of suspended graphene. *Nanoscale* **4**, 3065–3068 (2012).

[24] Engel, L., Lib, C.-C., Shahar, D., Tsui, D. & Shayegan, M. Microwave resonances in low-filling insulating phases of two-dimensional electron and hole systems. *Physica E* **1**, 111–115 (1997).

[25] Ye, P. D. *et al.* Correlation lengths of the Wigner-crystal order in a two-dimensional electron system at high magnetic fields. *Phys. Rev. Lett.* **89**, 176802 (2002).

[26] Chen, Y. *et al.* Microwave resonance of the 2D Wigner crystal around integer Landau fillings. *Phys. Rev. Lett.* **91**, 016801 (2003).

[27] Lewis, R. M. *et al.* Microwave resonance of the bubble phases in $1/4$ and $3/4$ filled high Landau levels. *Phys. Rev. Lett.* **89**, 136804 (2002).

[28] Chen, Y. P. *et al.* Evidence for two different solid phases of two dimensional electrons in high magnetic fields. *Phys. Rev. Lett.* **93**, 206805 (2004).

[29] Shklovskii, B. I. Coulomb gap and variable range hopping in a pinned Wigner crystal. *Phys. Stat. Sol. (c)* **1**, 46–50 (2004).

[30] Drichko, I. L. *et al.* Melting of Wigner crystal in high-mobility n-GaAs/AlGaAs heterostructures at filling factors $0.18 > \nu > 0.125$: Acoustic studies. *Phys. Rev. B* **94**, 075420 (2016).

[31] Gouttenoire, V. *et al.* Digital and FM demodulation of a doubly clamped single-walled carbon-nanotube oscillator: towards a nanotube cell phone. *Small* **6**, 1060–1065 (2010).
